# From mechanisms to therapeutics: molecular insights into gastrointestinal injury under high-altitude hypoxia

**DOI:** 10.3389/fmicb.2026.1707886

**Published:** 2026-02-06

**Authors:** Yonglu Yu, Yan Zhang, Yunsheng Yang

**Affiliations:** 1 Medical School of Chinese PLA, Beijing, China; 2 Microbiota Lab and Clinical Division of Microbiota, Department of Gastroenterology and Hepatology, The First Medical Center, Chinese PLA General Hospital, Beijing, China

**Keywords:** high altitude, hypoxia, intestinal injury, gut microbiota, probiotics

## Abstract

The extreme environmental conditions of a plateau have an important impact on the economic development of the area, including tourism and employment. High-altitude environments, characterized by hypoxia, low atmospheric pressure, and intense ultraviolet radiation, are recognized as key contributors to gastrointestinal injury. These environmental stresses promote oxidative stress, inflammatory responses, and gut microbiota dysbiosis, resulting in intestinal barrier disruption, increased permeability, and immune imbalance, which collectively predispose individuals to gastrointestinal disorders and multi-organ dysfunction. Accumulating evidence suggests that natural bioactive molecules, probiotics, and synbiotics exert protective effects against high-altitude-induced intestinal injury via diverse mechanisms. Accordingly, this review focuses on the key mechanisms of high-altitude hypoxia-induced intestinal injury and discusses the therapeutic potential of intestinal function-enhancing molecules. This work aims to offer a theoretical framework and identify potential intervention targets for the management of gastrointestinal disorders associated with high-altitude exposure.

## 1 Introduction

The high-altitude (> 2,500 m) environments represent a unique ecological system characterized by low atmospheric pressure, reduced oxygen partial pressure, cold temperatures, high wind speeds, increased evaporation, intense radiation, and highly variable climatic conditions. Among the environmental challenges at high altitude, hypobaric hypoxia has the most profound impact on human physiology. Both acute and chronic exposure to low oxygen and low atmospheric pressure can disrupt multiple physiological systems, leading to pathological organ dysfunction and disease. Common symptoms include dizziness, dyspnea, anorexia, impaired cognitive and behavioral performance, and gastrointestinal disturbances (Luks et al., 2017). The gastrointestinal tract is particularly susceptible to hypoxia and ischemia and is often the first organ system to be affected. Epidemiological data indicates a marked increase in the incidence of gastrointestinal disorders at elevations above 2,500 m (Fruehauf et al., 2020). Digestive symptoms such as nausea, vomiting, and loss of appetite are common manifestations of acute mountain sickness (AMS), affecting up to 81.4% of individuals who rapidly ascend to high altitudes, including short-term visitors and tourists (Anand et al., 2006; Kunin et al., 2020). Given the health needs of approximately 140 million permanent high-altitude residents, as well as specific populations such as climbers, field workers, and military personnel, the prevention and treatment of high-altitude gastrointestinal disorders have become an urgent medical priority.

As the largest immune and endocrine organ in the human body, the intestine plays a vital role in maintaining digestive function, energy homeostasis, and internal physiological balance through the integrity of its barrier function (Ahlman and Nilsson, 2001; Takiishi et al., 2017). Despite its essential role in maintaining physiological balance, the intestinal barrier is particularly susceptible to disruption under high-altitude hypoxic conditions, where oxidative stress, inflammatory cascades, and gut microbiota dysbiosis contribute to mucosal damage, immune imbalance, and gastrointestinal symptoms. Damage to the intestinal barrier increases gut permeability, allowing luminal contents to translocate across the epithelium. This microbial translocation and endotoxin release can activate systemic inflammatory responses via the gutgut sy axis. Consequently, the gut is increasingly recognized as the initiating organ in multi-organ dysfunction associated with high-altitude stress (Adak et al., 2014; Haitao et al., 2024). Recent advances in multi-omics and molecular biology have provided new insights into the mechanisms and interventions for high-altitude-associated gastrointestinal disorders. For example, the adaptive response of the intestinal mucosa to hypoxia is primarily coordinated by hypoxia-inducible factors (HIFs) (Yuan et al., 2024). Integrated metagenomic and metabolomic analyses have identified microbiota-derived butyrate as a protective factor in hypoxic adaptation (Zhao et al., 2024). Furthermore, microbiota-targeted synbiotic strategies, such as the combination of stachyose and Lactobacillus rhamnosus GG, have shown significant efficacy in hypoxic animal models by alleviating inflammation and preserving barrier integrity (Ren et al., 2024). This review focuses on the core mechanisms underlying hypoxia-induced intestinal injury at high altitude and summarizes current progress in the development of intestinal function-enhancing agents for use in hypoxic environments.

## 2 Oxidative stress: an initiating factor of hypoxia-induced injury

**FIGURE 1:**
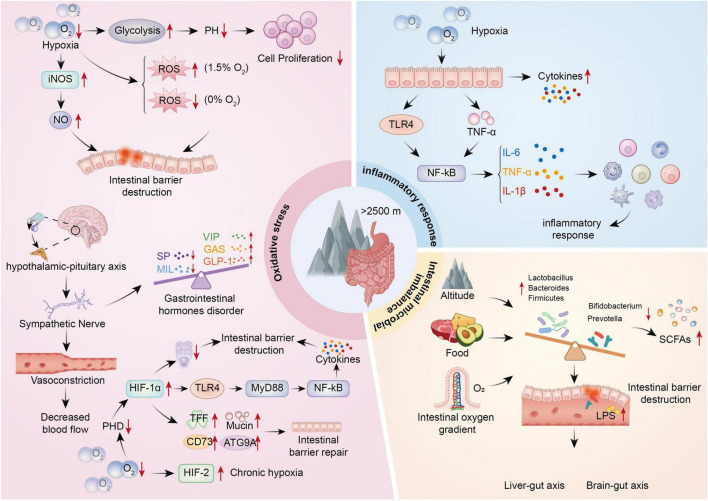
Mechanisms of hypoxia-induced intestinal injury at high altitudes.

Exposure to high-altitude environments introduces a complex array of physiological stressors, with hypobaric hypoxia being among the most prominent. These conditions challenge multiple systems in the human body, with the gastrointestinal tract being particularly vulnerable. Several interconnected mechanisms contribute to hypoxia-induced intestinal injury, including energy metabolism disruption, neuroendocrine dysregulation, and the complex bidirectional roles of HIF signaling (Figure 1).

### 2.1 Disturbance of energy metabolism

High-altitude hypoxia impairs aerobic metabolism, leading to enhanced glycolysis and intracellular acidosis. Prolonged exposure further disrupts mitochondrial structure, increases protein hydroxylation, and suppresses cell proliferation, ultimately contributing to mucosal injury and muscular layer atrophy in the gastrointestinal tract (Gruszczyk et al., 2022). In a study conducted during an extreme-altitude mountaineering expedition, enhanced cellular responses to hypoxia and oxidative stress were observed, along with activation of inflammatory pathways and impaired immune effector functions. During this process, the expression of glycolytic and antioxidant-related genes was concurrently upregulated. Metabolomic profiling of plasma samples also revealed significantly increased glutamine and fatty acid metabolism (Yin et al., 2025). Additional findings showed that hypoxia induces the expression of inducible nitric oxide synthase (iNOS), resulting in elevated nitric oxide (NO) production (Uusijärvi et al., 2015). While NO may confer tissue-protective effects under physiological conditions, excessive NO-particularly that derived from iNOS-acts as a potent oxidant and has been implicated in intestinal barrier disruption in high-altitude environments. At the same time, hypoxic exposure activates mitochondrial complex III and NADPH oxidase pathways, promoting the generation of reactive oxygen species (ROS). These ROS not only target epithelial membrane phospholipids but also damage thiol-containing structures within tight junction proteins such as occludin and ZO-1. In doing so, they compromise the physical barrier of the intestine and contribute to hypoxia-related signaling, including the stabilization of HIFs (Yin et al., 2025). Interestingly, recent evidence suggests that these oxidative stress effects are more prominent under moderate hypoxia (1.5% O2), whereas under extreme hypoxia (0% O2), intracellular ROS levels in intestinal epithelial cells actually decline (Gaur et al., 2021; Gruszczyk et al., 2022; Uusijärvi et al., 2015; Vissenaekens et al., 2019; Yin et al., 2025). This observation implies that severe hypoxic responses may be independent of mitochondrial ROS and possibly regulated by alternative suppressive mechanisms. Collectively, energy deficiency, pH imbalance, and ROS overproduction act synergistically to impair epithelial cell function and disrupt tight junction integrity, ultimately leading to intestinal barrier dysfunction.

### 2.2 Neuroendocrine dysregulation

High-altitude conditions such as cold, low atmospheric pressure, and hypoxia act as potent physiological stressors, activating systemic neuroendocrine and humoral responses that compromise gastrointestinal mucosal integrity and impair digestion and absorption (Lian et al., 2021; Zuhl et al., 2014). Acute exposure without adequate acclimatization rapidly activates the hypothalamus-pituitary-adrenal (HPA) axis. Enhanced sympathetic outflow opens mucosal arteriovenous shunts and increases catecholamine release, leading to vasoconstriction and a marked reduction in mucosal perfusion. This creates a vicious cycle of oxygen supply-demand imbalance, resulting in microvascular dysfunction, increased intestinal permeability, and impaired secretory function (Kocamaz et al., 2021). Sustained sympathetic overactivation also disrupts endocrine regulation, reducing digestive fluid secretion and altering the balance of gastrointestinal motility-related hormones. Notably, the levels of motilin (MIL) and substance P (SP) are markedly decreased, while vasoactive intestinal peptide (VIP), glucagon-like peptide-1 (GLP-1), and gastrin (GAS) are abnormally elevated. This hormonal imbalance not only impairs gastrointestinal motility but also reflects dysregulation of the neuroendocrine-gut axis under high-altitude stress (Hong et al., 2010).

### 2.3 Bidirectional regulation of the HIF signaling pathway

**FIGURE 2:**
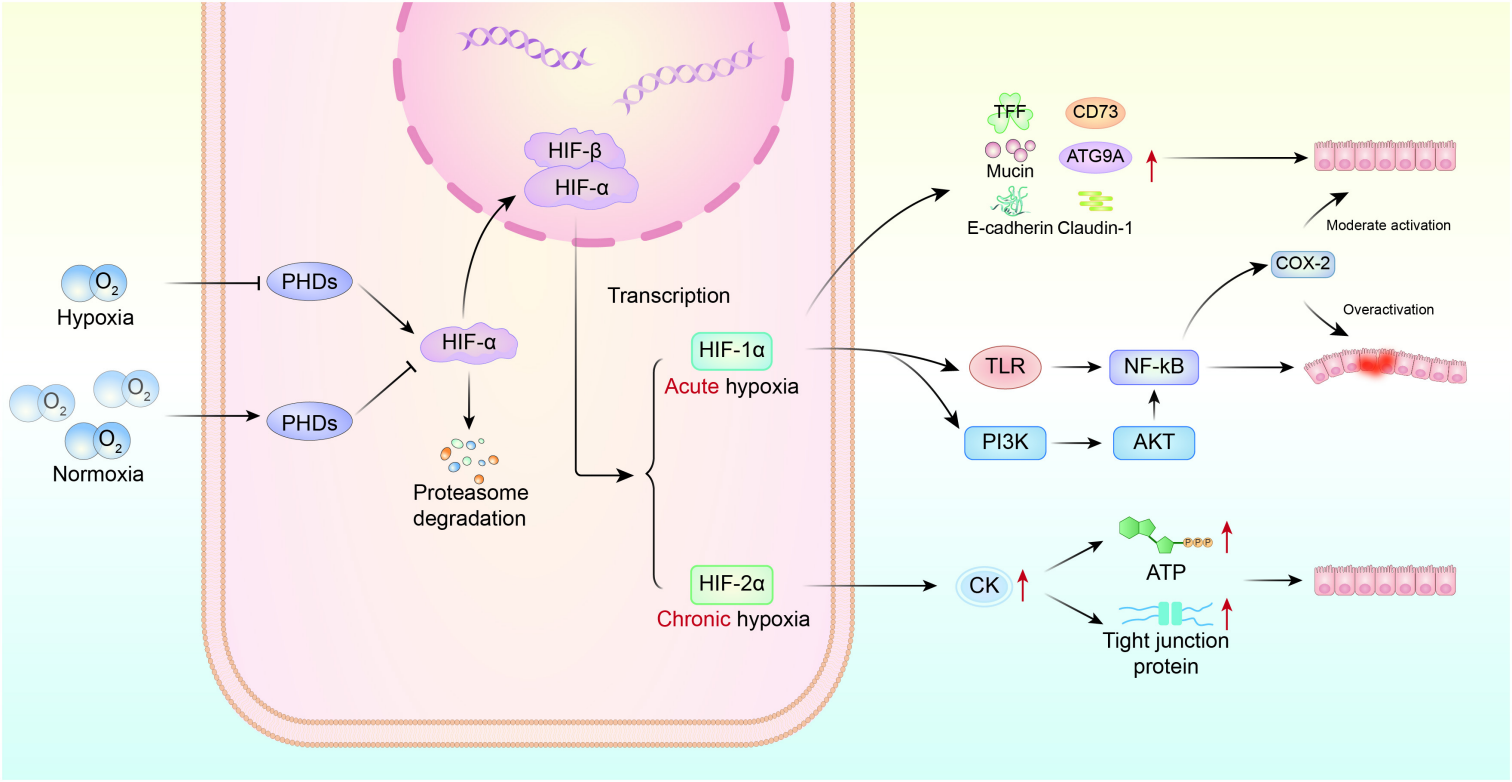
Role of HIF signaling pathway in intestinal injury induced by high-altitude hypoxia.

HIFs are central transcriptional regulators that mediate intestinal mucosal adaptation to hypoxic conditions. By modulating a wide range of metabolic, inflammatory, and barrier-related pathways, HIFs play a critical role in maintaining gut homeostasis (Figure 2) (Kim et al., 2020; Lian et al., 2021). Under normoxic conditions, HIF-1α is continuously targeted for degradation by prolyl hydroxylases (PHDs). In hypoxic environments, however, PHD activity is suppressed, leading to the stabilization of HIF-1α and subsequent activation of multiple downstream target genes. The role of HIF-1α in the intestine remains controversial. Although stabilized HIF-1α has been shown to enhance barrier integrity and mitigate inflammation by upregulating targets such as trefoil factors, CD73, E-cadherin, ATG9A, and various mucins (Dowdell et al., 2020; Hoffmann, 2021; Kim et al., 2020), other evidence suggests that under high-altitude hypoxia, excessive HIF-1α activation may induce iNOS expression and promote nitric oxide (NO) production, potentially improving microcirculation in the short term but contributing to redox imbalance over time (Wang et al., 2020; Zhang et al., 2015). Notably, HIF-1β (also known as ARNT), the constitutive partner of the HIF-1 heterodimer, is essential for the transcriptional activity of HIF-1α. Loss of HIF-1β disrupts this complex and impairs its downstream effects. In particular, HIF-1β deficiency has been shown to impair claudin-1 localization and compromise tight junction architecture, thereby exacerbating intestinal barrier damage (Saeedi et al., 2015). Experimental studies further confirm that HIF-1β-deficient mice exhibit more severe hypoxemia, greater weight loss, and increased cardiac workload under hypoxic exposure (Han et al., 2020). In addition to its protective roles, HIF-1α may also drive inflammation. It has been shown to bind directly to the TLR4 promoter, thereby activating the TLR4/MyD88/NF-κB signaling pathway, inducing the release of proinflammatory cytokines, and exacerbating mucosal injury (Kim et al., 2010; Xu et al., 2014a). HIF-1α expression is also regulated by the PI3K/AKT/NF-κB axis, and although basal COX-2 levels may be protective, excessive activation contributes to inflammation and epithelial disruption (Wan et al., 2014). A positive feedback loop between HIF-1α and NF-κB has been described: NF-κB promotes HIF-1α transcription in response to cytokines and oxidative stress, while HIF-1α in turn enhances NF-κB activation, amplifying inflammatory signaling and worsening barrier dysfunction (Zhang et al., 2012). Moreover, sustained activation of HIF-1α has been reported to reduce goblet cell numbers and impair their secretory function, further compromising mucosal barrier integrity (Zhang et al., 2025).

In contrast, HIF-2 appears to play a more targeted role in metabolic regulation. It modulates the creatine kinase (CK) gene family to maintain energy buffering capacity in intestinal epithelial cells. Under conditions of HIF-2 functional deficiency, CK expression is significantly reduced, disrupting energy homeostasis and exacerbating barrier dysfunction. Clinical studies have shown that patients with inflammatory bowel disease (IBD) display lower CK expression in intestinal biopsies, suggesting that impaired creatine metabolism may contribute to barrier loss (Glover et al., 2013). In mouse models, dietary creatine supplementation enhances the phosphocreatine/CK energy cycle and alleviates colitis, reinforcing the role of HIF-2 in supporting epithelial energy balance.

Interestingly, the expression kinetics of HIF isoforms differ: HIF-1α peaks within 4 h of hypoxia and subsequently declines, whereas HIF-2α peaks around 8 h and remains elevated for at least 24 h (Jaśkiewicz et al., 2022). These findings suggest that HIF-2 may play a more prominent role in long-term adaptation to chronic hypoxia, although the regulatory mechanisms governing the transition between HIF-1 and HIF-2 dominance remain to be elucidated.

## 3 Inflammatory response: an amplifier of hypoxia-induced intestinal injury

Hypoxia has increasingly been recognized as a potent trigger of inflammation. Both animal and clinical studies have demonstrated that even short-term hypoxic exposure can lead to the infiltration of inflammatory cells in multiple organs and upregulate circulating proinflammatory cytokines (Wang and Huo, 2021). Environmental stressors such as hypobaric pressure and hypoxia can induce systemic inflammatory responses by promoting the release of lipid mediators, including leukotrienes (LTs) and thromboxane A2 (TXA2). These mediators, in turn, activate coagulation cascades, disrupt hemostasis, and promote widespread thrombosis. As the body prioritizes perfusion to vital organs, intestinal blood flow is markedly reduced, rendering the gastrointestinal mucosa highly susceptible to hypoxic injury. Thus, the small intestine is considered one of the most hypoxia-sensitive organs (McKenna et al., 2022; Muza et al., 2010). At the cellular level, intestinal epithelial cells recognize microbial signals via Toll-like receptors (TLRs) and respond by secreting inflammatory mediators such as NF-κB, IL-6, TNF-α, and IL-1β (Cosin-Roger et al., 2017). Among these, NF-κB plays a central role in regulating inflammatory cascades. Under basal conditions, NF-κB remains sequestered in the cytoplasm through binding to its inhibitor IκB. During hypoxia, IκB is phosphorylated and degraded, allowing NF-κB to translocate into the nucleus and initiate transcription of proinflammatory genes (Bowser et al., 2018). *In vitro* studies using Caco-2 and T84 cells have demonstrated that hypoxic exposure directly upregulates TNF-α and IL-1β expression, further activating NF-κB signaling and amplifying downstream inflammatory response (Simmen et al., 2019).

Notably, the inflammatory consequences of hypoxia may vary with exposure severity and duration. Moderate hypoxia has been shown to attenuate intestinal inflammation by reducing mTOR-NLRP3 interaction and promoting autophagy activation (Yoo et al., 2021). In contrast, sustained hypoxia persistently activates the TLR4/NF-κB signaling pathway, leading to excessive release of cytokines and damage-associated mediators that compromise epithelial integrity. Moreover, bacterial translocation under hypoxic conditions can further aggravate barrier dysfunction by altering tight junction protein expression in a TLR-dependent manner (Wang and Huo, 2021). A study by Hill et al. provided additional insight, showing that low-pressure hypoxia combined with physical exercise increased intestinal permeability and MIP-1β expression, thereby elevating the risk of bacterial translocation. However, the systemic immune response appeared to be blunted, showing a shift toward anti-inflammatory signaling (Hill et al., 2020). Specifically, TNFα/IL-1RA and IL-1β/IL-1RA ratios were significantly reduced post-exercise, and phosphorylation of NF-κB in peripheral mononuclear cells was lower compared to normoxic controls, along with sustained downregulation of TNF-α protein levels. These findings highlight the complexity of hypoxia–inflammation interactions and underscore the need for further investigation into the contextual determinants of hypoxia-induced immune modulation.

## 4 Gut microbiota dysbiosis: a synergistic mechanism of hypoxia-induced intestinal injury

The high-altitude hypoxic environment can disrupt gut microbial homeostasis through multiple factors, including elevation, luminal oxygen gradients, dietary composition, and intestinal pH (Wang et al., 2022; Zuo et al., 2022). Increasing evidence highlights the gut microbiota as a key regulatory interface mediating host adaptation to high-altitude stress, influencing a variety of physiological responses such as energy metabolism, immune modulation, and oxygen utilization (Zhao et al., 2022; Zuo et al., 2022).

A multi-omics study involving 610 healthy young men revealed that with the migration of Han individuals from low to high altitude, their gut microbiota composition gradually shifted toward that of native Tibetans, and this shift was reversible upon returning to lower elevations (Han et al., 2024). Moreover, changes in host plasma metabolomic profiles and clinical parameters under high-altitude exposure were found to correlate significantly with alterations in gut microbial composition. Core taxa under hypoxic conditions may include Firmicutes, Akkermansia, Lactobacillus, and Prevotella (Bai et al., 2022). Metagenomic studies in animal models have demonstrated that hypobaric hypoxia increases Bacteroides abundance in feces, while Corynebacterium, Prevotella, and Coprococcus are reduced. Comparative population analyses showed that both Tibetan residents (T group) and long-term Han migrants (HH group) in Tibet exhibited higher levels of Firmicutes than lowland Han individuals (LH group), who instead showed enrichment of Bacteroidetes. At the genus level, Lactobacillus was elevated in the T group, whereas Prevotella was reduced; overall microbial diversity appeared lower in HH participants (Jia et al., 2020; Li and Zhao, 2015). In acutely exposed Han individuals, the abundance of Enterobacteriaceae, Gammaproteobacteria, and other potentially pathogenic taxa increased, indicating reduced microbial diversity and an elevated risk of opportunistic colonization (Li et al., 2016).

Clinical studies further support these findings. A randomized controlled trial found that individuals who developed acute mountain sickness (AMS) had a significantly higher relative abundance of Prevotella compared to non-AMS participants, suggesting that a reduced Bacteroides-to-Prevotella ratio may serve as a potential biomarker of hypoxia sensitivity (Karl et al., 2018). Similarly, a 47-day longitudinal assessment of seven mountaineers above 5,000 meters showed a marked decline in beneficial taxa such as Bifidobacterium, Corynebacterium, and Eggerthella, while Escherichia coli increased. These compositional shifts coincided with decreased serum IgA/IgM against LPS and elevated C-reactive protein (CRP), underscoring the link between microbial dysbiosis and systemic immune activation under high-altitude hypoxia (Kleessen et al., 2005; Li et al., 2022; Liu et al., 2021).

In addition to compositional shifts, the metabolic output of gut microbes plays a crucial role in altitude adaptation. Functional microbial metabolites such as short-chain fatty acids (SCFAs), volatile fatty acids (VFAs), essential amino acids, and vitamins contribute to host resilience under hypoxic stress (Geng et al., 2023). Notably, some studies have reported an increased abundance of butyrate-producing bacteria in the gut microbiota of high-altitude populations, which may represent an adaptive feature to enhance energy harvesting and barrier maintenance under hypoxic stress (Zhao et al., 2024). Plateau-endemic mammals, such as the plateau pika, harbor abundant SCFA-producing taxa, particularly members of the Lachnospiraceae and Clostridiaceae families, which facilitate enhanced energy harvesting and conversion of complex carbohydrates into SCFAs (Wang et al., 2022). Similar enrichment patterns have been observed in other high-altitude animals and humans. Metabolomic profiling of fecal samples from highland humans and pigs confirmed a greater SCFA synthesis potential compared to lowland counterparts (Zeng et al., 2020). SCFAs can exert anti-inflammatory effects by inhibiting NF-κB activation and histone deacetylase (HDAC) activity, thereby suppressing pro-inflammatory cytokine production (Hills et al., 2019; Shi et al., 2017). However, acute or severe hypoxic exposure can lead to a decline in butyrate-producing bacteria and Bacteroidetes, compromising SCFA synthesis, disrupting epithelial energy homeostasis, downregulating tight junction proteins, and ultimately impairing gut barrier integrity (Kleessen et al., 2005; Li et al., 2022; Liu et al., 2021). Thus, the balance between adaptive enrichment and stress-induced loss of SCFA producers is a crucial determinant of gut health at high altitude.

Microbial dysbiosis may also promote intestinal hyperpermeability, facilitating bacterial translocation and endotoxemia. High-altitude exposure has been associated with increased Enterobacteriaceae and E. coli abundance, accompanied by elevated CRP levels and reduced anti-LPS antibody titers (Li et al., 2022). Circulating lipopolysaccharide (LPS) can activate the TLR4/NF-κB pathway, amplifying inflammatory responses and damaging the intestinal mucosa (Bian et al., 2020; Luo et al., 2017). Barrier disruption further facilitates LPS absorption, leading to submucosal edema, villus epithelial cell necrosis, and increased permeability—establishing a vicious cycle of inflammation and tissue injury. In addition, microbe-derived metabolites such as LPS can propagate inflammatory signals systemically via the gut–liver and gut–brain axes, contributing to multi-organ dysfunction under extreme environmental stress (Li et al., 2025; Zhou et al., 2025).

## 5 Classification and recent advances of protective agents

### 5.1 Natural products and bioactive compounds from traditional Chinese medicine

Natural products can modulate multiple pathways involved in anti-inflammatory responses, oxidative phosphorylation, and antioxidative defense. Due to their strong antioxidant activity and favorable safety profiles, some natural compounds have been widely applied in the prevention and treatment of high-altitude hypoxia-related conditions. Berberine, a traditional treatment for gastroenteritis and diarrhea with thousands of years of clinical use, has been shown to alleviate IFN-γ and TNF-α-induced intestinal epithelial barrier damage by inhibiting the HIF-1α-mediated MLCK/pMLC signaling pathway and preserving the morphology and expression of tight junction proteins, although it exerts no significant effect on the NF-κB pathway (Cao et al., 2013). Paeoniflorin has demonstrated efficacy in repairing colonic epithelial injury by modulating serum metabolites and inhibiting the CDC42/JNK signaling pathway, thereby suppressing oxidative stress, inflammation, and apoptosis (Hu et al., 2024). Citrus segment membrane extract (CTPE), which is rich in pectin and flavonoids, has been shown to promote gut health. Recent studies report that CTPE significantly increases the abundance of Lactobacillus, upregulates the expression of tight junction protein mRNA and proteins, reduces serum D-lactate levels, and inhibits pro-inflammatory cytokines such as IL-6, TNF-α, and IFN-γ, thus effectively alleviating hypoxia-induced ileal injury in mice (Xue et al., 2024).

Non-steroidal anti-inflammatory drugs (NSAIDs), though widely used for inflammation control, are known to cause gastrointestinal damage with long-term use. Notably, their administration under high-altitude hypoxic conditions can exacerbate intestinal injury and disturb gut microbial homeostasis. Resveratrol (RSV) has been shown to mitigate NSAID-induced intestinal injury under high-altitude hypoxia by suppressing the TLR4/NF-κB/IκB signaling cascade, reducing IL-1β and TNF-α levels, increasing IL-10 expression, repairing mucosal barrier proteins, and enriching beneficial Clostridium species (Yu et al., 2023). Additionally, crude polysaccharides from Brassica rapa (BRP) significantly improved antioxidant enzyme activity, decreased inflammatory markers such as IL-6 and IL-1β, and enhanced the expression of claudin-1, occludin, and ZO-1 to restore barrier function in hypoxic mice. Combined chromatographic and metagenomic analysis further revealed that BRP increases butyrate levels, promotes the growth of beneficial bacteria including Akkermansia and Lactobacillus, and upregulates pathways for L-arginine biosynthesis II and L-methionine biosynthesis III, thereby enhancing antioxidant capacity (Liu et al., 2024). Recent studies have indicated that hypoxia downregulates low-density lipoprotein receptor-related protein 6 (LRP6) expression, whereas overexpression of LRP6 or its N-terminal domain can restore Wnt/β-catenin signaling inhibited by hypoxia and endoplasmic reticulum stress. Curcumin and allopurinol have been shown to target LRP6 to ameliorate intestinal barrier damage in DSS-induced colitis models (Liu et al., 2022).

Salidroside, the major antioxidant compound of Rhodiola, improves intestinal mucosal barrier function by inhibiting the TLR4/NF-κB signaling pathway, reducing intestinal permeability markers, and upregulating tight junction proteins. It also enhances mucosal immunity by increasing secretory IgA (sIgA), the proportion of CD4^+^T cells, and the CD4^+^/CD8^+^ ratio, thereby alleviating intestinal immune imbalance caused by hypoxic stress (Xu et al., 2023). In addition to its intestinal benefits, salidroside also exerts protective effects against high-altitude hypoxia-induced pulmonary and cerebral edema (Atochin et al., 2016; Shi et al., 2012). Due to its strong antioxidant properties and high safety, Rhodiola extracts have demonstrated promising potential in clinical applications for high-altitude hypoxia adaptation. While microbial fermentation and biotransformation in the gut are often essential for the absorption and activity of natural compounds, the mechanistic basis of these interactions remains incompletely understood.

### 5.2 Butyrate

The gut microbiota generates a variety of metabolites through fermentation of dietary fiber, including SCFAs, volatile fatty acids (VFAs), essential amino acids, and vitamins. Among these, butyrate—a key SCFA—has been increasingly recognized for its protective role in the intestine under high-altitude hypoxic conditions. Both *in vitro* and *in vivo* studies have demonstrated that butyrate preserves epithelial barrier integrity and suppresses inflammatory responses through multiple mechanisms. In a co-culture model of Caco-2 and RAW264.7 cells, sodium butyrate (1 mM) was shown to downregulate the NF-κB/NLRP3 pathway, reduce the secretion of proinflammatory cytokines such as IL-1β, TNF-α, and IL-8, and enhance the expression of tight junction proteins, thereby repairing LPS-induced epithelial permeability damage (Bakshi and Mishra, 2025). Similarly, in hypoxia-exposed porcine colonic epithelial cells, butyrate supplementation alleviated structural damage, an effect attributable not only to its nutritional role but also to SCFA-mediated intracellular signaling (Dengler et al., 2021). Single-cell transcriptomic analyses have further revealed that high-altitude hypoxia activates HIF-1α–mediated glycolysis, upregulates the expression of proinflammatory cytokines (e.g., IL-1β, IL-6, TNF-α), and suppresses anti-inflammatory factors (e.g., IL-10, TGF-β), thereby promoting M1 macrophage polarization and worsening barrier dysfunction. Butyrate has been shown to downregulate HIF-1α and key glycolytic enzymes (such as GCK, PFK, PKM, and LDH), correct macrophage polarization imbalance, and attenuate mucosal injury (Yan et al., 2024). Moreover, Mendelian randomization studies in Tibetan populations have provided supportive evidence at the population level, indicating that butyrate-related metabolic pathways are closely associated with downregulation of HIF-1α signaling. This mechanism may not only mitigate hypoxia-induced stress responses but also contribute to adaptive regulation of erythropoiesis (Zhao et al., 2024). Collectively, these findings highlight butyrate as a microbiota-derived signaling metabolite with pleiotropic effects on inflammation, energy metabolism, and epithelial integrity under hypoxic stress, making it a promising target for future therapeutic strategies in high-altitude gastrointestinal disorders.

### 5.3 Protective functional microbiota

Akkermansia muciniphila, a key mucin-degrading bacterium residing in the intestinal mucus layer, has been inversely associated with various metabolic and inflammatory disorders. Recent studies indicate that high-altitude hypoxia leads to a marked reduction in A. muciniphila abundance, while supplementation with either live bacteria or extracellular vesicles derived from this species can attenuate metabolic dysregulation and inflammation. Further studies have shown that A. muciniphila degrades intestinal mucus to generate short-chain fatty acids (SCFAs), which help maintain epithelial barrier integrity. It upregulates the expression of tight junction proteins, suppresses pro-inflammatory cytokines such as IL-6 and TNF-α, and inhibits NLRP3 inflammasome activity, collectively reducing intestinal permeability and mitigating hypoxia-induced mucosal injury. These findings underscore its potential as a next-generation probiotic for intestinal protection under high-altitude hypoxic conditions (Mishra and Bakshi, 2023).

Beyond the well-studied taxa, recent investigations have identified additional microbial players in high-altitude adaptation. For instance, Blautia A has been shown to exert anti-inflammatory effects and reinforce the intestinal barrier, thereby contributing to gut health and enhancing host acclimatization to hypoxia (Su et al., 2024b). Another emerging candidate, Faecalibacterium duncaniae, mitigates high-altitude-induced intestinal barrier damage by elevating levels of 2-ketoglutaric acid, a metabolite implicated in epithelial protection and metabolic regulation (Sun et al., 2025). These findings expand the repertoire of protective microbiota and highlight the potential of targeting specific microbial strains or their metabolic outputs for therapeutic intervention in high-altitude gastrointestinal disorders. Several altitude-adapted probiotic strains isolated from native populations have also demonstrated protective effects in hypoxic models. For example, Lactobacillus johnsonii YH1136, isolated from healthy Tibetan girls, was shown to restore microbial balance in mice by enriching beneficial lactobacilli and suppressing pathogenic genera such as Staphylococcus and Corynebacterium. This strain also activated miR-196a-1-3p and miR-3060-3p, which inhibit pathogen-induced disruption of barrier signaling pathways, thereby lowering gut permeability and preserving tight junction structure (Wan et al., 2022). Similarly, selenium nanoparticles (SeNPs) biosynthesized by Lactobacillus casei ATCC 393 were shown to alleviate oxidative stress–induced intestinal damage in a murine hypobaric hypoxia model. Oral administration of these SeNPs reversed villus injury, normalized intestinal DAO and MPO levels, enhanced tight junction expression in the ileum, and restored microbial homeostasis, thereby strengthening mucosal antioxidant defenses (Dou et al., 2023).

Beyond specific strains, certain functional taxa enriched in high-altitude environments may promote host metabolic resilience under hypoxia. Notably, short-term hypoxic exposure significantly reduces intestinal uric acid levels, a phenomenon negatively correlated with the abundance of Lachnospiraceae. Functional analysis revealed that this family can degrade uric acid efficiently via purine metabolism, thereby enhancing host energy adaptation and mitigating hypoxia-induced epithelial injury. Metagenomic profiling of long-term high-altitude residents further confirmed that sustained enrichment of Lachnospiraceae is linked to lower intestinal urate levels, supporting a potential role for these bacteria in modulating purine homeostasis and promoting physiological resilience in extreme environments (Su et al., 2024a).

The reoxygenation phase after high-altitude exposure appears to be a critical window for microbiota-driven metabolic reprogramming. During the first week of reoxygenation, tyrosine metabolism is rapidly activated, with its intermediate metabolites significantly correlated with both Rothia abundance and scores of high-altitude deacclimatization syndrome. Other genera such as Barnesiella, Parabacteroides, and Megasphaera were linked to circulating levels of L-arginine, sphingosine-1-phosphate (S1P), and α-D-glucose, together constituting a biomarker network involved in early metabolic adaptation. Hematological analyses during this period revealed decreased mean corpuscular hemoglobin concentration (MCHC) alongside elevated mean corpuscular volume (MCV) and red cell distribution width (RDW-SD), reflecting erythrocyte remodeling. Notably, the first month of reoxygenation represents a key period for reshaping microbiota α-diversity. This restructuring, potentially mediated by tyrosine metabolism–microbiota interactions, may drive antioxidant enzyme activation and facilitate recovery from hypoxia-induced metabolic stress (Zhao et al., 2025).

### 5.4 Synbiotics

Synbiotics, which integrate probiotics and prebiotics into a unified formulation, have emerged as a promising strategy for preserving intestinal homeostasis under high-altitude hypoxia by simultaneously modulating the microbiota, supporting host metabolism, and reinforcing epithelial barrier function. One study developed a synbiotic whey beverage by fermenting whey with 0.8% (w/v) Brassica rapa L. crude polysaccharides and kefir microbial inoculum. This formulation exhibited strong antioxidant capacity in mice, significantly reducing oxidative stress and preserving intestinal barrier function through attenuation of inflammation, preservation of mucosal architecture, increased goblet cell numbers, and reduced epithelial apoptosis. Additionally, it promoted the enrichment of beneficial taxa such as Intestinimonas and members of the Butyricicoccaceae family while suppressing potentially harmful genera such as Marvinbryantia and Proteus. The beverage also elevated levels of SCFAs, berberine, and nicotinic acid, activated AMPK signaling pathway, and altered niacin and nicotinamide-related metabolites associated with suppression of Marvinbryantia, thereby attenuating intestinal inflammation and barrier damage (Niu et al., 2024). In another study simulating a high-altitude hypoxic environment, the combination of stachyose (STA) and Lactobacillus rhamnosus GG (LGG) significantly increased intestinal SCFA levels, particularly butyrate. This promoted the expression of tight junction proteins in intestinal cells, reduced levels of IL-1β, TNF-α, and hypoxia-inducible factors, and enhanced the activity of antioxidant enzymes such as TGF-β, SOD, CAT, and GSH-Px. The synbiotic also reshaped microbial composition by lowering the Firmicutes/Bacteroidetes ratio and increasing genera associated with anti-inflammatory effects and SCFA production, offering a novel therapeutic strategy for gut dysfunction under high-altitude hypoxia (Ren et al., 2024). More notably, a synbiotic formulation containing a Bifidobacterium-Lactobacillus mixture and fructooligosaccharides was shown to modulate immune responses. It alleviated excessive activation of both innate and adaptive immunity by inhibiting Th17-skewed immune responses, reducing NK cell activity, and downregulating chemokines such as CXCL12/SDF-1α. At the same time, it improved the gut microbial environment by increasing beneficial genera like Lactococcus and Allobaculum, and reducing the abundance of pathogenic taxa such as Prevotella and Clostridium, thereby relieving intestinal mucosal barrier injury induced by low-pressure hypoxia (Khanna et al., 2021).

### 5.5 Other functional enhancers

In addition to natural compounds and microbiota-based interventions, several small-molecule nutrients and bioactive factors have shown potential protective effects on the gut under high-altitude hypoxic conditions.

Nitrate is a stable inorganic salt widely present in the human body and can be obtained through consumption of nitrate-rich vegetables and fruits. Studies have shown that nitrate supplementation under hypoxic conditions can effectively improve exercise performance (Macuh and Knap, 2021). Further research revealed that nitrate enhances the expression of intestinal tight junction proteins by promoting the nuclear translocation and transcriptional activity of HIF-1α, primarily through the EGFR/PI3K/AKT/mTOR signaling pathway. In animal studies, nitrate supplementation significantly attenuated hypoxia-induced weight loss and reduced serum biomarkers of intestinal injury, suggesting a systemic protective effect (Xu et al., 2024). However, a recent meta-analysis indicated that nitrate supplementation did not prevent acute mountain sickness (AMS) at high altitudes, and the evidence supporting its ergogenic effect remains limited (Nafi’an et al., 2024).

As supportive dietary agents for high-altitude adaptation, antioxidants have also been applied to some extent in managing hypoxia-induced intestinal dysfunction. A randomized, double-blind, placebo-controlled trial demonstrated that daily supplementation with a combination of antioxidants (1,000 mg vitamin C, 400 IU vitamin E, and 600 mg α-lipoic acid) significantly reduced the severity of AMS, as evidenced by lower Lake Louise scores, along with improved arterial oxygen saturation and increased caloric intake in the antioxidant group compared to placebo (Bailey and Davies, 2001). Although the sample size was limited and mechanistic insights were not deeply investigated, the findings suggest a potential role for antioxidant vitamins in physiological adaptation to hypoxia. Additional studies confirmed that vitamin E can alleviate hypoxia-induced organomegaly and intestinal villus damage, potentially via inhibition of the HIF and TLR4/NF-κB signaling pathways. Specifically, vitamin E downregulates HIF-1α, HIF-2α, and TLR4 expression in the ileum, suppresses NF-κB activation, reduces proinflammatory cytokine release, and upregulates the tight junction protein occludin, thereby mitigating oxidative stress (Xu et al., 2014a). Nevertheless, other trials reported that a combined antioxidant regimen containing ascorbic acid, α-tocopherol, and α-lipoic acid failed to reduce AMS incidence or improve oxygen saturation and pulmonary artery pressure in healthy individuals exposed to 5,200 meters of altitude (Baillie et al., 2009), indicating ongoing debate regarding the efficacy of antioxidant supplementation.

Exosomes, nanovesicles actively secreted by cells and enriched with proteins, RNA, DNA, and lipids, have gained attention in the context of hypoxia-related diseases. Studies have shown that exosomes derived from yak milk, enriched in bta-miR-34a, can activate HIF and apoptosis-related signaling pathways, significantly enhancing the survival rate (by up to 13%) of intestinal epithelial cells (IEC-6) under hypoxic conditions and preserving barrier integrity (Gao et al., 2021). Similarly, exosome-like nanoparticles derived from Robinia pseudoacacia flowers (RFELNs) can suppress HIF-1α/HIF-2α expression, inhibit NOX4/ALOX5-mediated ROS accumulation and lipid peroxidation, thereby reducing ferroptosis and alleviating gastric and intestinal mucosal injury. These nanoparticles also partially modulate hypoxia-induced microbiota and metabolic imbalances (Wang et al., 2024).

Glutamine is a critical nutrient for maintaining intestinal mucosal architecture, epithelial barrier function, immune modulation, and microbial homeostasis, playing a central role in preventing gut-derived infections and maintaining barrier integrity (Perna et al., 2019). Under high-altitude hypoxia, glutamine supplementation significantly enhances antioxidant enzyme activity, reduces the accumulation of oxidative stress markers such as malondialdehyde, and alleviates oxidative damage. Concurrently, it suppresses the release of IL-6 and TNF-α, upregulates tight junction protein expression, and maintains epithelial integrity, thus ameliorating hypoxia-induced mucosal injury. The protective mechanism is closely related to inhibition of the TLR4/MyD88/NF-κB signaling pathway (Xu et al., 2014b). Furthermore, some studies suggest that prophylactic glutamine administration may attenuate stress- and endotoxemia-induced increases in intestinal permeability and bacterial translocation, although its long-term efficacy remains to be confirmed (Abbasi et al., 2024) (Tables 1–3).

**TABLE 1:** Potential therapeutic agents for high-altitude hypoxia-induced intestinal injury.

Agent	Source	Proposed mechanism	Experimental dose (model)	Evidence level	References
Berberine	Coptis chinensis	Inhibits HIF-1α/MLCK pathway; enhances tight junction proteins	100 mg/kg (mice)	Preclinical	Cao et al., 2013
Paeoniflorin	Paeonia lactiflora	Suppresses CDC42/JNK pathway; reduces oxidative stress and apoptosis	50 mg/kg (mice)	Preclinical	Hu et al., 2024
Citrus tangerine pith extract (CTPE)	Citrus fruits	Enriches Lactobacillus; upregulates tight junctions; anti-inflammatory	400 mg/kg (mice)	Preclinical	Xue et al., 2024
Resveratrol (RSV)	Grapes, berries	Mitigates NSAID-induced injury via TLR4/NF-κB suppression; enriches Clostridium	50 mg/kg (rats)	Preclinical	Yu et al., 2023
*Brassica rapa* L. polysaccharide (BRP)	Turnip	Enhances antioxidant enzymes; increases butyrate and beneficial bacteria (Akkermansia, Lactobacillus)	400 mg/kg (mice)	Preclinical	Liu et al., 2024
Sodium butyrate	Gut microbiota/Dietary fiber	Inhibits NF-κB/NLRP3; corrects macrophage polarization; enhances barrier	1 mM (cells); 150 mg/kg (mice)	Preclinical	Bakshi and Mishra, 2025; Dengler et al., 2021
Akkermansia muciniphila	Human gut	Degrades mucin to SCFAs; suppresses NLRP3; upregulates tight junctions	10^8^ CFU/day (mice)	Preclinical	Mishra and Bakshi, 2023
Lactobacillus johnsonii YH1136	Tibetan population	Modulates miR-196a-1-3p/miR-3060-3p; inhibits pathogen-induced barrier disruption	10^9^ CFU/day (mice)	Preclinical	Su et al., 2024b
Synbiotic (Stachyose + LGG)	Synthetic	Increases butyrate; enhances antioxidant enzymes; reduces inflammation	STA 0.5 g/kg + LGG 10^9^ CFU (mice)	Preclinical	Ren et al., 2024
Salidroside	Rhodiola rosea	Inhibits TLR4/NF-κB; enhances sIgA and CD4^+^/CD8^+^ ratio	20 mg/kg (rats)	Preclinical	Xu et al., 2023
Nitrate	Dietary vegetables	Promotes HIF-1α nuclear translocation via EGFR/PI3K/AKT/mTOR	0.1 mM (cells); 1 mM in drinking water (mice)	Preclinical	Macuh and Knap, 2021; Xu et al., 2024
Vitamin E + C + α-lipoic acid	Synthetic/Dietary	Antioxidant; inhibits HIF/TLR4/NF-κB pathways	400 IU E + 1,000 mg C + 600 mg ALA (human)	Clinical trial (RCT)	Bailey and Davies, 2001
Glutamine	Synthetic/Dietary	Inhibits TLR4/MyD88/NF-κB; enhances antioxidant enzymes	0.75 g/kg (rats)	Preclinical	Xu et al., 2014b

**TABLE 2:** Practical strategies for preventing GI injury under high-altitude hypoxia.

Agent	Specific measure	Proposed mechanism
Dietary modifications	Increase dietary fiber (whole grains, legumes, vegetables)	Promotes gut microbiota to produce protective short-chain fatty acids (e.g., butyrate), enhancing barrier function.
	Consume nitrate-rich foods (spinach, beetroot)	Enhances tight junction protein expression via the HIF-1α pathway.
	Consider citrus peel extracts (e.g., tangerine pith)	Rich in flavonoids; exerts anti-inflammatory and antioxidant effects, enriches *Lactobacillus*.
Probiotics and synbiotics	Supplement with lactobacilli (e.g., *L. rhamnosus* GG) and Bifidobacteria	Modulates gut microbiota, enhances barrier integrity, and suppresses inflammation.
	Use Synbiotics (e.g., Fructooligosaccharides + Lactobacilli)	Synergistically enhances probiotic colonization and production of beneficial metabolites like butyrate.
	Consider next-gen probiotics (e.g., *Akkermansia muciniphila*)	Degrades mucin to produce SCFAs, strengthens barrier, and inhibits NLRP3 inflammasome.
Natural extracts	Rhodiola (contains Salidroside)	Inhibits TLR4/NF-κB pathway, enhances secretory IgA (sIgA), and improves immune balance.
	Berberine	Inhibits HIF-1α/MLCK signaling pathway, protecting tight junction proteins.
	Curcumin	Targets LRP6 to restore Wnt/β-catenin signaling, improving barrier function.
Supplemental nutrients	Glutamine	Inhibits TLR4/MyD88/NF-κB signaling, boosts antioxidant enzymes, and maintains barrier.
	Sodium Butyrate	Suppresses NF-κB/NLRP3 pathway, corrects macrophage polarization, and reinforces barrier.
	Combined Antioxidants (Vit C + E + α-Lipoic Acid)	Inhibits HIF/TLR4/NF-κB pathways, reducing oxidative stress.
Behavioral and pharmacological caution	Avoid non-essential NSAIDs (e.g., Ibuprofen)	NSAIDs exacerbate intestinal mucosal injury and disrupt gut microbiota under hypoxia.
	Ascend gradually and avoid strenuous exercise upon arrival	Reduces sympathetic overactivation and consequent gut ischemia.
	Maintain body warmth, avoid cold stress	Cold exposure exacerbates sympathetic response, reducing intestinal blood flow.

**TABLE 3:** Glossary of the molecules.

Abbreviation	Full form	Abbreviation	Full form
AMS	Acute mountain sickness	MIL	Motilin
β-catenin	Beta-catenin	MIP-1β	Macrophage inflammatory protein-1 beta
BRP	Brassica rapa polysaccharide	MLCK	Myosin light chain kinase
CDC42	Cell division cycle 42	MPO	Myeloperoxidase
CD	Cluster of differentiation	mTOR	Mechanistic target of rapamycin
CK	Creatine kinase	NF-κB	Nuclear factor kappa B
COX-2	Cyclooxygenase-2	NLRP3	NLR family pyrin domain containing 3
CRP	C-Reactive protein	NO	Nitric oxide
DAO	Diamine oxidase	PI3K	Phosphoinositide 3-kinase
EGFR	Epidermal growth factor receptor	PHD	Prolyl hydroxylase
GLP-1	Glucagon-like peptide-1	RCT	Randomized controlled trial
GSH-Px	Glutathione peroxidase	ROS	Reactive oxygen species
HDAC	Histone deacetylase	RSV	Resveratrol
HIF	Hypoxia-inducible factor	SCFA	Short-chain fatty acid
HPA	Hypothalamus-pituitary-adrenal	SeNPs	Selenium nanoparticles
IBD	Inflammatory bowel disease	sIgA	Secretory immunoglobulin A
IFN-γ	Interferon gamma	SOD	Superoxide dismutase
iNOS	Inducible nitric oxide synthase	TGF-β	Transforming growth factor beta
JNK	c-Jun N-terminal kinase	TLR4	Toll-like receptor 4
LGG	Lactobacillus rhamnosus GG	TNF-α	Tumor necrosis factor alpha
LPS	Lipopolysaccharide	VIP	Vasoactive intestinal peptide
LRP6	Low-density lipoprotein receptor-related protein 6	Wnt	Wingless-related integration site

## 6 Prospects and challenges

In high-altitude environments, extreme ecological conditions often lead to reduced host resistance and increased susceptibility to gastrointestinal diseases. Multiple interrelated mechanisms, such as oxidative stress, inflammatory cascades and gut microbiota dysbiosis, contribute to the disruption of intestinal barrier integrity and lead to both localized and systemic pathological responses. However, current preventive and therapeutic strategies targeting altitude-associated gastrointestinal disorders remain limited and show suboptimal clinical efficacy. Emerging studies have highlighted the bidirectional regulatory role of HIFs, which coordinate energy metabolism, inflammatory responses, and tight junction protein expression, thereby mediating both mucosal adaptation and injury. Activation of the downstream TLR4/NF-κB signaling axis under acute hypobaric hypoxia has been implicated in barrier disruption and microbial translocation. In recent years, increasing attention has been given to gut microbiota and its metabolites as key mediators of host immune modulation, antioxidant defense, and metabolic homeostasis. These functions position them as critical efficacy-enhancing elements in the context of high-altitude adaptation. Interventions involving natural products, glutamine, exosomes, and antioxidants have demonstrated protective effects on the intestinal barrier by targeting critical signaling pathways such as HIF and TLR4/NF-κB. These findings offer diverse strategies for maintaining intestinal function and barrier integrity under hypoxic conditions. Despite encouraging advances, major challenges remain. The underlying mechanisms are not fully understood, clinical translation is limited, and systematic evaluation is lacking. Most current findings are based on animal models, with insufficient data on human heterogeneity, long-term safety, and applicability. Effective prevention and management of altitude-induced gastrointestinal dysfunction are essential for safeguarding health, supporting regional development, and ensuring the sustainability of high-altitude tourism. Future research must therefore expand in both depth and breadth. In terms of mechanistic depth, it is essential to leverage advanced biological models such as organoids and organ-on-a-chip systems, combined with cutting-edge technologies like single-cell sequencing and spatial transcriptomics, to systematically elucidate the fine-tuned regulatory networks of key signaling pathways under hypoxia, particularly host-microbiota interactions, thereby accelerating target screening and mechanistic validation. In terms of research breadth, it is imperative to move beyond the current population framework and extend the scope to more diverse subpopulations. This should include ethnic groups with different genetic adaptation backgrounds, as well as special populations such as individuals with pre-existing gastrointestinal conditions or those frequently exposed to high altitudes due to occupational demands, such as military personnel, mountaineers, and construction workers. Concurrently, in-depth exploration of the influences of sex, age, and hormonal status on susceptibility is warranted. Such efforts will ultimately enable a shift from population-wide to individualized precision interventions, laying a solid scientific foundation and providing effective health safeguards for the prevention and management of high-altitude-related gastrointestinal diseases and their comorbidities.

## 7 Expert opinion

Research on gastrointestinal injury under high-altitude hypoxic conditions has yielded encouraging progress in recent years, particularly in the elucidation of molecular mechanisms and the development of interventional strategies. Current studies have not only identified key targets such as the HIF signaling pathway, TLR4/NF-κB axis, and butyrate metabolism, but also revealed that natural compounds like salidroside and berberine, as well as microbial agents including Akkermansia muciniphila and specific Lactobacillus strains, exhibit significant gut-protective effects in animal models. These findings provide a theoretical foundation for developing functional foods, dietary supplements, or adjunctive drugs aimed at mitigating high-altitude gastrointestinal disorders, with potential applications for high-altitude workers, mountaineers, and long-term residents. However, the translation of these findings into clinical practice faces multiple challenges. Most studies remain at the preclinical stage, lacking large-scale, multi-center randomized controlled trial (RCT) evidence. The heterogeneity of high-altitude environments further complicates the standardization and personalization of interventions. Moreover, the stability, delivery efficiency, and long-term safety of these formulations require further validation, while limited policy and financial support collectively hinder the rapid application of these achievements. Additional bottlenecks must be addressed. Mechanistically, many studies remain descriptive, focusing on phenotypic observations and preliminary pathway validation without in-depth exploration of dynamic changes in HIF isoforms or detailed molecular interactions between gut microbiota and the host. Existing animal models also fail to fully recapitulate the complex physiological responses of humans under hypoxic conditions, particularly the integrated regulation of the neuro-endocrine-immune-microecological network. Technologically, integration and analysis of multi-omics data remain inadequate, and there is a lack of efficient, systematic tools for deciphering system-level mechanisms of the “gut-organ axis.” Furthermore, current studies often suffer from insufficient sample representation regarding ethnicity, sex, age, and baseline health status, limiting the generalizability of the conclusions. To address these issues, future research should incorporate more physiologically relevant models such as organoids and organ-on-a-chip systems, combined with advanced technologies like single-cell sequencing, spatial transcriptomics, and metabolic flux analysis to enhance mechanistic depth and precision. Concurrently, more cohort studies involving diverse populations should be conducted to establish big data-driven predictive models of high-altitude adaptability. Despite these challenges, the research prospects in this field remain promising. The interaction between gut microbiota and the host remains one of the most valuable directions in high-altitude medicine. Integrating multi-omics technologies with artificial intelligence is expected to advance “precision high-altitude medicine,” enabling personalized interventions. Additionally, the roles of the “gut-brain axis” and “gut-liver axis” in high-altitude stress responses warrant further exploration. In contrast, single-target antioxidant or anti-inflammatory strategies may gradually be replaced by multi-target, integrated intervention approaches due to their inconsistent efficacy and limited scope. With the accumulation of clinical data and the application of novel delivery systems, gut-protective products based on probiotics/synbiotics or plant extracts are expected to enter the market. Simultaneously, assessment tools evaluating high-altitude adaptability using gut microbiota and metabolic biomarkers may be preliminarily applied for screening high-risk populations. In the future, “gut health assessment” may become a routine part of pre-altitude examinations, and AI-powered personalized nutrition and microecological intervention strategies could be incorporated into high-altitude disease prevention guidelines, facilitating a paradigm shift from reactive treatment to proactive prevention.
